# Frequency and Severity of Thrombocytopenia in Neonatal Sepsis

**DOI:** 10.7759/cureus.22665

**Published:** 2022-02-27

**Authors:** Maria Arabdin, Adnan Khan, Sikandar Zia, Sarbiland Khan, Gulrukh S Khan, Maryam Shahid

**Affiliations:** 1 Department of Pathology, Rehman Medical College, Peshawar, PAK; 2 Department of Pediatrics, Rehman Medical Institute, Peshawar, PAK; 3 Clinical Haematology, Kingston Hospital NHS Foundation Trust, London, GBR; 4 Department of Medicine, Rehman Medical Institute, Peshawar, PAK; 5 Department of Physiology, Khyber Medical College, Peshawar, PAK

**Keywords:** critical care, neonatal intensive, thrombocytopenia, sepsis, neonates

## Abstract

Background

Neonatal sepsis includes numerous systemic illnesses such as septicemia, meningitis, urinary tract infections, and pneumonia. In developing countries, the major reason for neonatal mortality is septicemia, which accounts for almost 50% of overall deaths. Thrombocytopenia is one of the most common hematological problems during the neonatal period, affecting the majority of sufferers admitted to the neonatal intensive care unit (NICU). The aim of our study was to find the frequency of thrombocytopenia and its severity in neonates with sepsis.

Methods

The study was conducted at the Department of Hematology at Khyber Medical University, Peshawar, Pakistan. A total of 170 neonates with an age of fewer than 28 days, both genders, and positive blood cultures were included in the study using a non-probability consecutive sampling technique. Data was recorded in predesigned questionnaires after taking informed consent. Data were recorded and analyzed using SPSS version 26 (IBM Corporation, Armonk, NY, USA).

Results

Of the 170 neonates, 104 (61.2%) were males, with a mean age of 12.12±8.88 days. The majority of the babies 73 (42.9%) were in the age group of 0-7 days. Most of the neonates 72 (42.4%) were born via normal vaginal delivery (NVD). Of the neonates, 117 (68.82%) presented with fever, and 105 (61.76%) were reluctant to feed. Furthermore, 65.29% of the neonates had thrombocytopenia, of which 34 (20%) had mild, 43 (25.3%) had moderate, and 34 (20%) had severe thrombocytopenia. In neonates with positive blood culture, the platelet level was low (p<0.001). In the case of gram-negative organisms, the level of platelets was lower as compared to gram-positive organisms (p<0.001).

Conclusion

Sepsis is still a common cause of newborn thrombocytopenia. The fact that it is present in more than half of all culture-positive sepsis episodes indicates the severity of the condition. This condition is further defined by higher percentages of early-onset gram-negative septicemia compared to gram-positive sepsis.

## Introduction

Neonatal sepsis incorporates diverse systemic ailments such as septicemia, meningitis, urinary tract infections, and pneumonia [1]. In developing international locations, the primary cause of neonatal death is septicemia, accounting for almost 50% of general deaths [2,3]. Its miles assessed that sepsis develops in 20% of neonates, of which 1% die in the early days [4]. The reported incidence for sepsis ranges from 1 to 10 per 1000 live births, but studies based on a huge population are rare, and the available data mostly is aimed at infants with high risk such as premature or very low birth weight (VLBW) infants in developed countries [5]. Based on the onset, sepsis is divided into three categories: early-onset sepsis (fewer than three days of age), late-onset sepsis (LOS) (at 3-28 days of age), and late late-onset sepsis (at 29-120 days of age). Among all three types, late-onset sepsis (LOS) is frequent, particularly in VLBW neonates. One of the early indicators for neonatal sepsis is thrombocytopenia [6].

Thrombocytopenia is one of the most common hematological disorders at newborn age, affecting the majority of neonates admitted to the NICU [7]. Neonates admitted to NICUs develop thrombocytopenia in 20%-35% of all admissions, and a hike in percentage is noted with a drop in gestational age [8,9]. The majority of neonates present with mild to moderate thrombocytopenia. Sepsis in the newborn is one of the chief causes of thrombocytopenia in neonates, and it can become very severe and can increase the risk of bleeding within 24 hours after developing an infection [10].

The exact mechanism of low platelets in neonatal sepsis is unknown, but it has been proposed that sepsis causes endothelial injury, which in turn triggers the reticuloendothelial system. Platelet consumption exceeds production and causes thrombocytopenia [11]. Thrombocytopenia and neonatal sepsis associations have been highlighted by recognizing thrombocytopenia as the prominent key risk feature for sepsis-related fatalities in neonates [12].

An observational study by Noreen et al. showed that the frequency of thrombocytopenia is higher in neonates admitted to the NICU for any reason [13]. Neonatal sepsis must not be considered as a homogenous entity, as it spurns both the pathogenic and clinical variations among numerous causative microorganisms and clinical syndromes and features of septicemia. No local study has been done in the recent past that emphasizes the incidence of thrombocytopenia in neonatal sepsis. In this research, we will present the characteristics of thrombocytopenia related to sepsis in both gram-negative and gram-positive cells. This will be beneficial for early diagnosis and treatment, predicting morbidity and mortality. The results of this study will provide us with local statistics on the magnitude of neonatal thrombocytopenia in sepsis, and this will provide a window for further research.

## Materials and methods

This was a descriptive cross-sectional study conducted at the Department of Hematology at Khyber Medical University, Peshawar, Pakistan. The sample size was calculated according to the WHO formula by taking the prevalence of thrombocytopenia in neonates as 20% [12], a margin of error of 6%, and a confidence interval of 95%. A non-probability consecutive sampling technique was adopted. All neonates less than 28 days old, of both genders, and positive blood culture were included. Those with a mother’s history of ITP, SLE, or other autoimmune disorders on medication during pregnancy (sulfonamides, quinine, quinidine, thiazides, tolbutamide, vancomycin, hydralazine, and heparin) and neonates with a history of bleeding disorders in the family, trisomies, or Turner/Noonan’s syndromes were excluded.

The study was to commence after approval was obtained from the Advanced Studies and Research Board and ethical board of Khyber Medical University, Peshawar, Pakistan. The aim of our investigation was explained to all parents/guardians, and informed written consent was taken as well. Patient information such as demographics and clinical examinations was recorded on a purposefully designed questionnaire. Blood samples were collected from each patient by a trained nurse, and the samples were sent to the laboratory for full blood count estimation. The platelet count was less than 150×109/L. A peripheral smear was made, and platelet count and morphology were checked manually. Erythrocyte sedimentation rate (ESR) was also estimated manually. C-reactive protein (CRP) was analyzed from plasma via Cobas 601 (Roche, Basel, Switzerland) and was expressed in mg/dL.

SPSS version 26 (IBM Corporation, Armonk, NY, USA) was used to record and analyze data. Descriptive statistics were used to calculate the mean and standard deviation of all numerical variables, such as age, hemoglobin level, white blood cell count, platelet count, CRP level, and ESR, while categorical variables (gender, blood culture, age group, mode of delivery, presenting symptoms, and blood group) were presented in percentage and frequency. For the comparative analysis of sociodemographic data, hematological parameters, infective markers, and blood culture, with the severity of thrombocytopenia, ANOVA was used. A p-value of 0.05 was kept as significant.

## Results

The demographic characteristics of the neonates are given in Table 1. Of the 170 neonates, 104 (61.2%) were males and 66 (38.8%) were females. The majority of the neonates (73, 42.9%) were in the age group of 0-7 days. The overall mean age of the neonates was 12.12±8.88 days; the mean age for males was 11.18±7.790 days, while for females, it was 13.62±10.13 days. The majority of the neonates 81 (47.6%) were in the weight group between 2.5 and 3.5 kg. Most of the neonates 72 (42.4%) were born via normal vaginal delivery (NVD).

**Table 1:** Demographic characteristics of the neonates admitted to the NICU (n=170)

Variable	Frequency	Percentage (%)
Gender		
Male	104	61.2
Female	66	38.8
Age group		
0–7 days	73	42.9
8–14 days	31	18.2
15–21 days	29	17.1
22–28 days	37	21.8
Weight		
2.5–3.5 kg	81	47.6
3.6–4.5 kg	76	44.7
>4.5 kg	13	7.6
Mode of delivery		
Normal vaginal delivery	72	42.4
Cesarean section	49	28.8
NVD with episiotomy	49	28.8
Feeding		
Mother breastfeed	112	65.9
Formula milk	27	15.9
Both/mix	31	18.2

The majority of the mothers experienced no infection during pregnancy. However, only 58 experienced some sort of infection, of which 29 (17.1%) experienced a flu-like illness, 22 (12.9%) acute gastroenteritis, and seven (4.1%) other infections, including chest infection and genital infection. Table 2 shows the hematological and infective markers of the neonates.

**Table 2:** Mean hematological and infective markers of the neonates admitted to the NICU (n=170) CRP: C-reactive protein, ESR: erythrocyte sedimentation rate

Parameters	Hemoglobin (g/dL)	White blood cell (10^9^/L)	Platelet count (cells/L)	Neutrophils (%)	Lymphocytes (%)	CRP (mg/dL)	ESR (mm/hour)
Mean	13.114	10.701	182.947	43.251	41.821	4.588	21.076
Standard deviation	2.094	15.636	156.894	10.998	10.461	7.062	14.168
Minimum	7.80	4.39	2	13.50	8.20	0	9
Maximum	21.21	21.70	589	78.10	81	45	98

Blood culture was positive in 114 (61%) of the participants and was negative in 56 (39%). Furthermore, in positive blood culture, the organisms isolated were gram-negative in 77 (67.54%) and gram-positive in 27 (23.68%). In this study, of the 170 total neonates, 111 (65.29%) had thrombocytopenia, of which 34 (20%) had mild (platelets: 101-150 cells/L), 43 (25.3%) had moderate (platelets: 50-100 cells/L), and 34 (20%) had severe thrombocytopenia (platelets: >50 cells/L) (Table 3).

**Table 3:** Distribution of thrombocytopenia in the neonates admitted to the NICU (n=111)

Thrombocytopenia	Frequency	Percent (%)
Mild (101–150 cells/L)	34	20
Moderate (50–100 cells/L)	43	25.3
Severe (<50 cells/L)	34	20
Normal	59	34.7

While comparing blood culture results with thrombocytopenia, it was observed that out of 111, 92 (82.88%) had positive blood cultures, in which 26 (23.42%) had mild thrombocytopenia, 34 (30.63%) had moderate thrombocytopenia, and 32 (28.82%) had severe thrombocytopenia.

In gram-positive cases, three (3.26%) had mild thrombocytopenia, 12 (13.04%) had moderate thrombocytopenia, four (4.34%) had severe thrombocytopenia, and in gram-negative cases, 23 (25%) had mild thrombocytopenia, 22 (23.91%) had moderate thrombocytopenia, and 28 (30.43%) had severe thrombocytopenia.

By doing comparative analysis by applying ANOVA, it was noted that the age group was statistically significant, with a p-value of 0.010, which is less than 0.05. The lower the age group, the lower the platelet level. However, the other parameters were not significant (p>0.05): gender, p=0.548; weight, p=0.346; mode of delivery, p=0.160; maternal risk factors, p=0.756; fetal risk factors, p=0.096; and feeding, p=0.65 (Table 4).

**Table 4:** Comparative analysis of the sociodemographic data with the severity of thrombocytopenia (n=111) df: degree of freedom, F: variation, Sig: significance

Sociodemographic factors	Sum of squares	df	Mean square	F	Sig
Age	885.559	3	295.186	3.931	0.010
Gender	0.510	3	0.170	0.708	0.548
Weight	1.941	3	0.647	1.112	0.346
Mode of delivery	3.601	3	1.200	1.744	0.160
Maternal risk factor	3.511	3	1.170	0.396	0.756
Fetal risk factor	13.361	3	4.454	2.153	0.096
Feeding	1.005	3	0.335	0.538	0.657

Similarly, by doing the comparative analysis of hematological, infective, and blood cultures, it was found that it was significant in the case of C-reactive protein; the higher the CRP level, the lower the platelet level, with a p-value of 0.001. Similarly, in the case of blood culture, it was noted that, with positive blood culture, the platelet level was lower (p<0.001). Regarding the isolate, it was noted that, in the case of gram-negative organisms, the level of platelets was lower as compared to gram-positive organisms (p<0.001) (Table 5).

**Table 5:** Comparative analysis of hematological parameters, infective markers, and blood culture with the severity of thrombocytopenia (n=111) df: degree of freedom, F: variation, Sig: significance

Hematological parameters, infective markers, and blood culture	Sum of squares	df	Mean square	F	Sig
Hemoglobin	24.218	3	8.073	1.868	0.137
White blood cells	711.604	3	237.201	0.970	0.409
Neutrophils	268.515	3	89.505	0.736	0.532
Lymphocytes	213.628	3	71.209	0.647	0.586
C-reactive protein	1835.711	3	611.904	15.402	0.000
Erythrocyte sedimentation rate	1271.044	3	423.681	2.154	0.095
Blood culture	15.701	3	5.234	35.208	0.000
Organism isolated	17.895	3	5.965	14.967	0.000

## Discussion

Thrombocytopenia is a common complication of neonatal sepsis and is one of the most prognostic independent causes of sepsis-related death. Every year, over 1.6 million newborn deaths occur worldwide, with 40% of them occurring in poorer nations, primarily Asia and Africa. This condition is a prevalent concern in neonates with confirmed sepsis, with thrombocytopenia happening in 66% of the total cases in our cross-sectional study. The results of our study are in good correlation with a cohort study, where 20% had severe onset of the disease, and the results revealed an almost fourfold increase in mortality in neonatal sepsis with thrombocytopenia [12]. Earlier, Charoo et al. investigated 200 VLBW infants with sepsis, and thrombocytopenia was detected in 61.5% of them [14]. The increased incidence of thrombocytopenia in VLBW newborns may be due to a partial response to thrombocytopenia in terms of platelet and thrombopoietin production, mostly during sepsis with diminished energy reserves in the host [15].

The use of vascular catheters is strongly linked to intravascular thrombosis. Catheters can enhance platelet consumption [16]. They ultimately lead to vascular wall damage mechanically by altering the flow of blood, but they can also contain possibly thrombogenic agents or be used to inject toxic chemicals into the vascular walls [17]. In our study, males were more affected. This is in accordance with the study of Fanaroff et al., who found that the incidence of sepsis is considerably higher in male newborns than in female ones [18].

The severity of thrombocytopenia differs depending on the causative bacteria. In this study, gram-negative sepsis had a considerably higher rate of early thrombocytopenia than gram-positive sepsis. In 30.43% of gram-negative sepsis episodes, there was severe early thrombocytopenia, while in gram-positive sepsis, only 4.34% were investigated for severe thrombocytopenia. This finding supports a prior study that found that thrombocytopenia was more common in gram-negative sepsis [19]. The likely mechanisms of thrombocytopenia associated with gram-negative sepsis are augmented destruction of platelets by absorption, antibody-mediated binding, and activation. In animal models, cell-free extracts comprising lipopolysaccharide and a constituent of gram-negative bacteria’s cell wall have been demonstrated to cause thrombocytopenia [20].

According to our findings, blood culture and the degree of thrombocytopenia differ significantly in gram-positive versus gram-negative sepsis. Because the mainstream of our study sample had a positive blood culture, we assumed that this was due to the underlying distribution of bacteria. Previous research on the relationship between the kind of gram stain and the prevalence of thrombocytopenia in newborn sepsis has produced mixed results. When relating infections with gram-positive and gram-negative bacteria in neonates with extremely low birth weight, Manzoni et al. found no variance in the occurrence of thrombocytopenia, but they employed a different thrombocytopenia threshold [21]. Others, on the other hand, have observed similar results to ours and claim that thrombocytopenia is more common in gram-negative sepsis [22,23]. However, the size of the sample was typically limited, and outcomes were derived generally from populations of neonates with low birth weight and premature infants. Our findings are based on a large, recent sample of sepsis-affected infants from a variety of gestational ages. Because gram-negative sepsis is more severe than gram-positive sepsis and because sepsis can cause dispersed intravascular clotting, thrombocytopenia may simply be a sign of the severity of sepsis. In neonatal sepsis, an undeviating pathophysiological mechanism of endotoxins formed by gram-negative bacteria could play a role. Both bacterial groupings have a lot of inhomogeneity, with each bacterial species having varying degrees of pathogenicity [24].

In our findings, when sociodemographic data were compared with the severity of thrombocytopenia, only the age group was found to be statistically significant. Similarly, while comparing hematological, infectious, and blood complete counts, it was discovered that in the case of C-reactive protein, the greater the CRP level, the lower the platelet level. This finding was also supported by Rabindran et al., in which they studied the CRP with platelet level in neonates and found out that the greater the CRP, the higher the thrombocytopenia [25]. Furthermore, the severity of gram-negative sepsis increased.

Thrombocytopenia should be considered as the first sign of sepsis, even before the rise of infective markers such as CRP and raised TLC in neonates. If a neonate presents with clinical signs and symptoms of sepsis with thrombocytopenia, management should be started even if infective markers are within the normal range.

## Conclusions

In our study, we found that in neonatal sepsis, more than half of the neonates developed thrombocytopenia and 20% developed severe thrombocytopenia. It was independently associated with gram-negative sepsis, low gestational age, and high CRP. To conclude, it is critical to screen for and treat thrombocytopenia in all babies brought to the NICU, including those who appear to be at low risk, because the incidence and mortality linked with this illness are significant. Because sepsis is still a common cause of newborn thrombocytopenia and the severity of thrombocytopenia in sepsis varies from mild to moderate to severe, the fact that thrombocytopenia is present in more than half of sepsis cases with positive blood cultures indicates the severity of the condition.
